# The Slavic *NBN* Founder Mutation: A Role for Reproductive Fitness?

**DOI:** 10.1371/journal.pone.0167984

**Published:** 2016-12-09

**Authors:** Eva Seemanova, Raymonda Varon, Jan Vejvalka, Petr Jarolim, Pavel Seeman, Krystyna H. Chrzanowska, Martin Digweed, Igor Resnick, Ivo Kremensky, Kathrin Saar, Katrin Hoffmann, Véronique Dutrannoy, Mohsen Karbasiyan, Mehdi Ghani, Ivo Barić, Mustafa Tekin, Peter Kovacs, Michael Krawczak, André Reis, Karl Sperling, Michael Nothnagel

**Affiliations:** 1 Department of Clinical Genetics, Institute of Biology and Medical Genetics, 2^nd^ Medical School, Charles University, Prague, Czech Republic; 2 Institute of Medical and Human Genetics, Charité-Universitätsmedizin Berlin, Germany; 3 Department of Informatics, 2^nd^ Medical School, Charles University, Prague, Czech Republic; 4 Department of Pathology, Brigham and Women’s Hospital, Harvard Medical School, Boston, Massachusetts, United States of America; 5 DNA Laboratory, Department of Pediatric Neurology, 2^nd^ Medical School, Charles University and University Hospital Motol, Prague, Czech Republic; 6 Department of Medical Genetics, The Children's Memorial Health Institute, Warsaw, Poland; 7 Department of Bone Marrow Transplantation and Cancer Immunotherapy, Hadassah-Hebrew University Medical Center, Jerusalem, Israel; 8 National Genetics Laboratory, University Hospital of Obstetrics and Gynecology, Medical University, Sofia, Bulgaria; 9 Max Delbrück Center for Molecular Medicine (MDC), Berlin-Buch, Germany; 10 Institute of Human Genetics, Martin-Luther University Halle-Wittenberg, Halle, Germany; 11 Tanz Centre for Research in Neurodegenerative Diseases, University of Toronto, Toronto, Canada; 12 Department of Paediatrics, University Hospital Center Zagreb and University of Zagreb, School of Medicine, Zagreb, Croatia; 13 Division of Pediatric Molecular Genetics, Ankara University School of Medicine, Ankara, Turkey; 14 Leipzig University Medical Center, IFB AdiposityDiseases, Leipzig, Germany; 15 Institute of Medical Informatics and Statistics, Christian-Albrechts University, Kiel, Germany; 16 Institute of Human Genetics, Friedrich-Alexander-Universität Erlangen-Nürnberg (FAU), Erlangen, Germany; 17 Cologne Center for Genomics, University of Cologne, Köln, Germany; National Cancer Institute, UNITED STATES

## Abstract

The vast majority of patients with Nijmegen Breakage Syndrome (NBS) are of Slavic origin and carry a deleterious deletion (c.657del5; rs587776650) in the *NBN* gene on chromosome 8q21. This mutation is essentially confined to Slavic populations and may thus be considered a Slavic founder mutation. Notably, not a single parenthood of a homozygous c.657del5 carrier has been reported to date, while heterozygous carriers do reproduce but have an increased cancer risk. These observations seem to conflict with the considerable carrier frequency of c.657del5 of 0.5% to 1% as observed in different Slavic populations because deleterious mutations would be eliminated quite rapidly by purifying selection. Therefore, we propose that heterozygous c.657del5 carriers have increased reproductive success, i.e., that the mutation confers heterozygote advantage. In fact, in our cohort study of the reproductive history of 24 NBS pedigrees from the Czech Republic, we observed that female carriers gave birth to more children on average than female non-carriers, while no such reproductive differences were observed for males. We also estimate that c.657del5 likely occurred less than 300 generations ago, thus supporting the view that the original mutation predated the historic split and subsequent spread of the ‘Slavic people’. We surmise that the higher fertility of female c.657del5 carriers reflects a lower miscarriage rate in these women, thereby reflecting the role of the *NBN* gene product, nibrin, in the repair of DNA double strand breaks and their processing in immune gene rearrangements, telomere maintenance, and meiotic recombination, akin to the previously described role of the DNA repair genes *BRCA1* and *BRCA2*.

## Introduction

Nijmegen Breakage Syndrome (NBS [MIM: 251260]) is a chromosome instability disorder characterized by microcephaly, growth retardation, immunodeficiency, hypersensitivity to X-irradiation, and an exceptionally high risk for lymphoid malignancy [1–3]. The *NBN* gene (also abbreviated *NBS*, *NBS1*, *ATV*, or *AT-V1*, amongst others) was identified as the cause of NBS in 1998 [4–6] and is located on chromosome 8q21 (GRCh38.p2: 89,933,336–89,984,733). The corresponding gene product, nibrin, is part of the trimeric MRE11/RAD50 complex that is involved in the repair of DNA double strand breaks (DSBs) and in their processing in immune gene rearrangements, telomere maintenance, and meiotic recombination (summarized in [7, 8]). This multi-functionality explains the complex NBS phenotype, particularly the very high incidence of malignancies that form the major cause of early death amongst NBS patients [2]. The majority of NBS patients originates from Eastern Europe and is homozygous for a common founder mutation in the *NBN* gene, namely a deletion of nucleotide positions 657 to 661 of the coding sequence in exon 6 (abbreviated as c.657del5; rs587776650) that leads to two truncated fragments, p26- and p70 nibrin [9].

To the best of our knowledge, *NBN* c.657del5 is the only Slavic founder mutation of high prevalence described so far. It obviously predated the medieval migration of the Slavic tribes. Until recently, our insights into the history of the Slavs, the largest population in Eastern and Southeastern Europe, were mainly based upon linguistic and archaeological evidence. Although a common origin of the Slavs is generally accepted, controversy exists about their original homeland and the timing of their historical diversification. During the early medieval waves of migration into Europe in the 5^th^ and 6^th^ century, the Slavs split into the Eastern Slavs (Belarusians, Russian, Ukrainians), the Western Slavs (e.g. Czechs, Poles, Slovaks, Sorbs), and the Southern Slavs (e.g. Bulgarians, Croats, Serbs, Slovenes) (cited in [10, 11]). A recent study of autosomal, mitochondrial and Y-chromosomal data coined the existence of two sub-groups that presumably existed some 1900 years ago, namely the East-West Slavs (from Poland to the Volga river) and the South Slavs (confined to the Balkan Peninsula) [12]. Moreover, the study revealed pronounced genetic similarities between the Czechs and their immediate Germanic neighbors while, further north, the Sorbs and the surrounding Germans were clearly genetically distinct [13]. However, the time of the initial division of the proto-Slavic tribes was not determined. From the evolutionary history of Y-chromosomal STR haplotype R1a, the Russian Plain branch (including proto-Slavic tribes) living in present-day Russia, Ukraine, Belarus, Poland and the Baltic countries is estimated to have appeared about 4,600 years ago [14].

Interestingly, a high frequency of heterozygous c.657del5 carriers has been reported from the Slavic populations of Poland, the Czech Republic/Slovakia (henceforth abbreviated as CS), the Ukraine, and Germany [15, 16]. Notably, heterozygous carriers were particularly prevalent among cancer patients in these populations (reviewed in [17, 18]), an association that was rigorously confirmed in a family-based study carried out with the Index-Test Method [19].

Whilst heterozygous carriers seemingly reproduce, despite their increased cancer risk, no parenthood of a homozygous individual has been reported so far. The high frequency of c.657del5 carriers in different Slavic populations therefore seems enigmatic and difficult to explain by genetic drift alone. This can readily be illustrated by a simple selection model with relative fitness values of 1-hs and 1-s for hetero- and homozygous mutation carriers [20]. Assuming s>0.9 and an initial mutation frequency of 0.1%, the latter is expected to decrease slightly (to ~0.085%), rather than increase, within 200 generations if h = 0. Moreover, the mutation frequency is bound to fall below 10^−11^ over the same time period if h = 0.1, i.e. if heterozygous carriers are of slightly reduced reproductive fitness (data not shown).

In the present study, we provide evidence from a family-based cohort study for a reproductive advantage of female heterozygous c.657del5 carriers that potentially explains the high frequency of this mutation in Eastern Europe by balancing selection. In addition, we estimated c.657del5 to be approximately 4000 to 6650 years old, using a large collection from unrelated carriers of Eastern, Western and Southern Slavic origin.

## Materials and Methods

### Reproductive success of *NBN* c.657del5 carriers

We assessed the relative reproductive success of heterozygous *NBN* c.657del5 carriers relative to their non-carrier relatives. To this end, we drew upon a large collection of families of NBS patients from the Czech Republic and Slovakia that were originally recruited as part of a study to assess the relative cancer risk of carriers and non-carriers [19]. For more than 20 years, one of us (E.S.) has seen virtually all patients with clinically confirmed NBS in these countries. The present study, more specifically, includes 24 families with 344 members for whom comprehensive medical records were available to us. Seven of these families had been counseled and repeatedly interviewed over 10–20 years whilst another seven were regularly contacted for over 20–25 years. The remaining 10 families were diagnosed and followed between 1994 and 2003. Molecular testing after 1998 confirmed that all NBS cases were homozygous for the *NBN* c.657del5 mutation.

Starting from the affected child in each pedigree (‘index patient’), as many relatives as possible were contacted through the parents of the index patient. Attempts were made to contact all grandparents, their siblings (if alive), aunts, uncles and cousins who lived in the Czech Republic. Relatives were recruited without prior knowledge of their c.657del5 carrier status. Of the 490 individuals contacted, 344 agreed to participate in our study.

Upon informed consent, oral interviews were conducted via structured questionnaires administered by one and the same person (E.S.). The questionnaires covered socio-demographics (including reproductive history), general medical history, life style and occupational history. Socioeconomic status was inferred from the educational level attained when leaving school. Family history and gynecological as well as obstetric history were recorded for female participants only. The vast majority of interviews took place at the homes of participants and required around 60 minutes each. Obligate heterozygotes were not included so that the interviewer was consistently ‘blinded’ with respect to the carrier status of their interviewees. After the interview, a blood sample was taken to assess the *NBN* germline mutation c.657del5 genotype [21].

We compared the number of offspring of c.657del5 carriers and non-carriers, separately for males and females. Statistical analyses were carried out with Fisher´s exact or an unpaired *t*-test, as appropriate.

### Age estimation for *NBN* c.657del5

#### Sampling of individuals

We used the family data available to us to reconstruct the original *NBN* c.657del5 haplotype and to estimate the age of the deletion. In addition, we analyzed Guthrie cards from newborn screening programs:, including 1502 samples from Bulgaria, 994 from Croatia, and 1035 from Lusatia (Germany), the latter being part of an extensively phenotyped sample from Eastern Germany [13]. We also asked colleagues for additional samples from NBS patients of Eastern, Western, and Southern Slavic origin (Table 1).

**Table 1:** Individuals and haplotypes used for the c.657del5 age estimation and ancestral haplotype inference.

Origin	Sampled individuals (heteroz.)	c.657del5 carrying chromos.	Unambiguously inferred c.657del5 carrying haplotypes
Austria	1 (1)	1	0
Belgium	1	2	0
Bulgaria	4 (4)	4	0
Czech Republic/Slovakia (CS)	9 (1)	17	11
Croatia	1	2	2
Germany (Germans)	22 (2)	42	4
Germany (Sorbs from Lusatia)	10 (10)	10	3
Italy	1	2	2
Poland	35	70	64
Russia	10 (1)	19	0
Turkey	3	6	6
Ukraine	6	12	0
*Total*	*103 (19)*	*187*	*92*

Haplotypes were inferred from both homo- and heterozygous deletion carriers.

#### Genotyping and reconstruction of haplotypes

Some 84 homozygous and 19 heterozygous *NBN* c.657del5 carriers were genotyped at markers covering a 5.7 Mb region around the deletion on chromosome 8q21. More specifically, 13 microsatellites located between D8S271 and D8S270 were analyzed (Table 2) in addition to 12 SNPs (Table 3). For 187 chromosomes carrying *NBN* c.657del5 (S1 File), 92 haplotypes could be deduced without ambiguity using the available family data (Table 1). These haplotypes formed the basis of our attempt to estimate the age of the mutation. The unambiguous haplotypes and the most likely albeit ambiguous haplotypes are listed in the Supporting Information (S1 File).

**Table 2:** Microsatellite markers and one InDel polymorphism used in this study.

Marker	Available alleles	Sample heterozygosity	Physical distance [kb]	Genetic map [cM]	
				Rutgers	Decode
D8S271	89	0.614	0	0	0
D8S273	85	0.494	393.2	0.0000000001	0.0000000001
rs6150693^‡^	84	0.023	1,020.1	0.6485	0.453
D8S1800	91	0.423	1,176.7	0.7300	0.5100
AFM289	87	0.130	1,586.4	0.8152	0.6662
H3GT	89	0.189	1,947.2	0.8903	0.8038
H2CA	89	0.209	2,307.9	0.9653	0.9414
D8S88	92	0.257	2,330.6	0.9700	0.9500
*NBN* c.657del5	92	0.000	2,440.2	1.1128	1.0357
H5CA	82	0.137	2,602.0	1.3235	1.1621
D8S1811	91	0.146	2,714.5	1.4700	1.2500
D8S1724	92	0.181	2,901.6	1.4700000001	1.5700
D8S1146	82	0.181	3,504.9	1.8017	1.7019
D8S1618	85	0.134	3,986.7	2.0665	1.8073
D8S270	86	0.716	4,502.3	2.35	1.92

All markers are located on chromosome 8q21. Physical distances according to human genome assembly hg19 (Genome Reference Consortium GRCh37).

^‡^Note that the InDel polymorphism rs6150693 (position 89607483), which affects 12 base-pairs, has been removed from dbSNP on Oct 17, 2013, due to mapping or clustering errors (http://www.ncbi.nlm.nih.gov/SNP/snp_ref.cgi?type=rs&rs=rs6150693), but is listed in UCSC’s Genome Browser (http://genome.ucsc.edu/).

**Table 3:** Additional single-nucleotide polymorphism markers at the 92 unambiguously inferred haplotypes in this study.

Marker	Available alleles	Allele on haplotype	Global MAF	Physical distance [kb]	Genetic map [cM]	
					Rutgers	Decode
rs10111232	90	C	0.0004	2,394.3	1.0529	0.9998
rs1063045	92	T	0.3792	2,429.3	1.0986	1.0272
rs1805794	92	G	0.3570	2,437.4	1.1092	1.0335
*NBN* c.657del5 (rs587776650)	92	c.657del5	-	2,440.2	1.1128	1.0357
rs2234744	92	T	0.3530	2,449.5	1.1249	1.0430
rs709816	92	T	0.3914	2,452.7	1.1291	1.0455
rs1061302	92	C	0.3528	2,465.3	1.1455	1.0553
rs3736639	92	A	0.3792	2,472.3	1.1546	1.0607
rs1063053	92	T	0.3219	2,476.8	1.1605	1.0643
rs13272541	92	C	<0.0002	2,493.4	1.1821	1.0772
rs1000249	86	A	<0.0002	2,554.3	1.2614	1.1248
rs6994202	86	A	0.2899	2,577.8	1.2920	1.1432
rs11994308	86	C	0.0899	3,142.2	1.6022	1.6226

All markers are located on chromosome 8q21. Physical distances according to human genome assembly hg19 (Genome Reference Consortium GRCh37). All SNPs were found to be monomorphic in our sample. **MAF:** minor allele frequency, obtained from dbSNP (http://www.ncbi.nlm.nih.gov/snp).

#### Genetic marker distances

We considered two commonly used genetic maps, namely the Rutgers map [22–25] and the high-resolution recombination map from deCODE Genetics [26]. We abstained from involving the genetic map provided by the Marshfield Clinic [27] because it does not resolve large parts of the investigated chromosomal region. Where possible, we used sex-averaged maps. If the available map information did not include genetic distances for particular markers, as was the case for some microsatellites and all SNPs, these distance were interpolated assuming proportionality between physical and genetic distance, and by drawing upon the closest marker of known genetic location on either side of an uncharted marker. Since each marker had to be assigned a unique location for the age estimation software to function properly (see below), we introduced an artificial genetic distance of 10^−9^ cM between adjacent markers if they had the same genetic location according to the map in question (Table 2).

#### Age estimation

We used the DMLE+ software [28] for estimating the age of *NBN* c.657del5. This software employs a Bayesian algorithm to infer the location and/or age of a founder mutation based upon patterns of linkage disequilibrium and associated demographic parameters [29]. Numerical integration in DMLE+ is done by Monte Carlo Markov Chain (MCMC). Note that this software does not estimate the time to the most recent common ancestor (MRCA), but the actual age of the mutation. We initially used DMLE+ assuming a known location of the mutation (Table 2) together with the following parameters: population growth rate 0.01, mutation population frequency 1.0 (since all haplotypes carried the mutation); burn-in iteration 5,000,000; data iterations 10,000,000; number of histogram bars 200; mutation age limits 0–5000 generations; no star-like genealogy; no sequence weights. Subsequently, we considered population growth rates of 0.005 and 0.001 as well in order to assess the effect of this parameter on the age estimate. The R statistical software [30], version 3.2.2, was used for descriptive statistics and for creating graphs. Perl was used for scripting the analysis with DMLE+.

Our study was approved by the Ethics Committee of the 2^nd^ Medical School of Charles University and University Hospital Motol, Prague, Czech Republic. All participants gave signed informed consent, with the exception of the Sorb probands [13]. The respective study was approved by the Ethics Committee of the Leipzig University, Germany, and all participants had given their signed informed consent.

## Results

### Reproductive success of *NBN* c.657del5 carriers

The reproductive success of female *NBN* c.657del5 carriers was compared to that of their non-carrier relatives in a cohort of 40 heterozygotes and 208 homozygotes for the wild-type allele. The average age was almost the same in the two groups (51.2 vs. 50.9 years, respectively). However, the average number of offspring differed significantly (P = 0.02), with 3.03 children born by carriers and 2.36 children born by non-carriers (Table 4). For an age-stratified analysis, we split the cohort into three age groups: <40 years, 41–60 years, and >60 years at the time of the last interview. In all three groups, carriers had more children on average than non-carriers, and this difference was statistically significant in the 41–60 years age group (Table 4). There was no statistically significant difference with respect to school and university attendance between carriers and non-carriers. Furthermore, we also compared the menstrual history of female carriers and non-carriers, characterized by the age at first menstrual period and the age at onset of menopause (females 30 years and older). We observed no significant difference between the two groups in this regard (data not shown). No significant difference in the number of offspring was observed between male mutation carriers and non-carriers (Table 5). Data on survival until reproductive age were not available to us.

**Table 4:** Number of offspring of female *NBN* c.657del5 carriers and non-carriers of different age groups.

Age group	Carriers of c.657del5				Non-carriers of c.657del5				P
	N	Average age	Number of offspring	Ratio	N	Average age	Number of offspring	Ratio	
**<41**	12	32.3	21	1.75	77	32.2	123	1.60	0.59
**41–60**	17	51.6	62	3.65	61	51.3	160	2.62	0.01
**>60**	11	71.2	38	3.45	70	71.0	207	2.96	0.47
**All groups**	40	51.2	121	3.03	208	50.9	490	2.36	0.02

Average age is given in years. The two-tailed P-values were obtained from unpaired t-test.

**Table 5:** Number of offspring of male *NBN* c.657del5 carriers and non-carriers of different age groups.

Age group	Carriers of c.657del5				Non-carriers of c.657del5				P
	N	Average age	Number of offspring	Ratio	N	Average age	Number of offspring	Ratio	
**<41**	20	31.5	34	1.70	62	34.9	109	1.76	0.80
**41–60**	21	50.7	61	2.90	66	51.3	158	2.39	0.25
**>60**	18	70.1	51	2.83	40	71.2	99	2.48	0.45
**All groups**	59	50.1	146	2.47	168	50.0	366	2.18	0.20

Average age is given in years. The two-tailed P-values were obtained from unpaired t-test.

The number of spontaneous or induced abortions could be ascertained for 32 of the 40 female carriers and for 145 of the 208 female non-carriers, with both subgroups being of almost the same average age (54.5 and 53.4 years). The relative number of induced abortions was virtually the same whilst the rate of spontaneous abortions was slightly albeit non-significantly lower among carriers (Table 6). The 48 obligate heterozygous females (not included in the above analysis) had 57 living offspring (30 affected by NBS), three fetuses diagnosed with NBS, and 11 pregnancies that were interrupted for other reasons. Two fetuses were lost spontaneously. Of 73 registered pregnancies, only two thus ended spontaneously.

**Table 6:** Reproductive histories for the subset of female *NBN* c.657del5 carriers and non-carriers who were older than 30 years, ascertained by the index test method and had information on the number of spontaneous or induced abortions available.

	Carriers of c.657del5	Non-carriers of c.657del5	P
**Number of mothers**	32	145	
**Average age (years)**	54.5	53.4	
**All registered pregnancies**	120	436	
**Number of living offspring** (proportion of all pregnancies)	102 (85.0%)	351 (80.5%)	0.46
**Number of spontaneous abortions** (proportion of all pregnancies)	7 (5.8%)	41 (9.4%)	
**Number of induced abortions** (proportion of all pregnancies)	11 (9.2%)	44 (10.1%)	

The two-tailed P-values were obtained from Fisher´s exact test.

### Age of the *NBN* c.657del5 mutation

**Fig 1:**
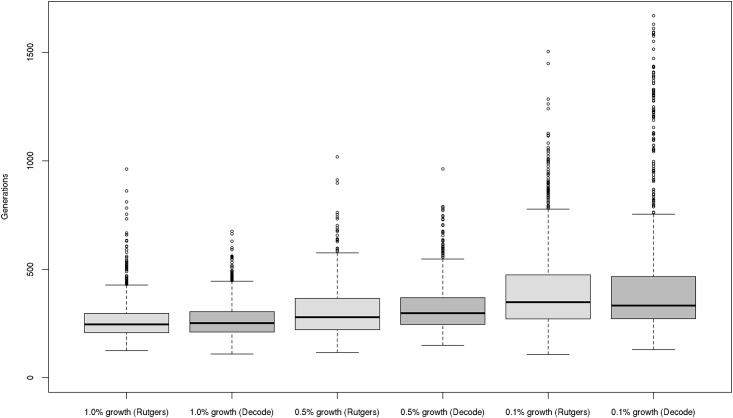
Posterior distribution of age estimates for *NBN* c.657del5. Box plots of the posterior age distribution refer to a population growth of 1.0%, 0.5% and 0.1%, respectively, and two genetic maps.

We applied the DMLE+ software to a set of microsatellite markers to estimate the age of *NBN* c.657del5. In order to assess the sensitivity of these analyses to the underlying assumptions, we assumed two different genetic maps and three different population growth rates. With an average population growth of 1% per generation, the mean posterior age estimate was 265 and 267 generations, respectively, for the Rutgers and the deCODE genetic map. This would roughly correspond to approximately 4000 to 6650 years, depending upon the presumed generation time (15 to 25 years; Table 7). Larger estimates of 343 to 369 and 509 to 698 generations were obtained for growth rates of 0.5% and 0.1%, respectively. These figures would correspond to an age range of 7000 to 12,500 years, assuming a generation time of 20 years (Table 7 and Fig 1).

**Table 7:** Age estimates for the *NBN* c.657del5 deletion.

	Years per generation	Mean (95% credible interval)	
		Rutgers map	Decode map
**Assumed population growth of 1.0%**			
**Generations**		264.8 (149.5–512.4)	267.2 (143.9–475.5)
**Years**	15	3972.0 (2241.8–7686.1)	4008.0 (2158.5–7131.8)
	20	5296.0 (2989.1–10,248.2)	5344.0 (2878.1–9509.1)
	25	6620.0 (3736.4–12,810.2)	6680.0 (3597.6–11,886.4)
**Assumed population growth of 0.5%**			
**Generations**		369.1 (186.4–702.0)	343.0 (185.4–759.2)
**Years**	15	5536.5 (2796.3–10,530.2)	5145.0 (2781.3–11,387.7)
	20	7382.0 (3728.4–14,040)	6860.0 (3708.4–15,183.6)
	25	9227.5 (4660.5–17,550.4)	8575.0 (4635.5–18,979.6)
**Assumed population growth of 0.1%**			
**Generations**		508.8 (166.9–1470.8)	698.0 (185.3–2261.6)
**Years**	15	7632.0 (2504.0–22,062.0)	10,470.0 (2779.3–33,924.3)
	20	10,176.0 (3338.6–29,416.0)	13,960.0 (3705.7–45,232.4)
	25	12,720 (4173.3–36,770.0)	17,450.0 (4632.1–56,540.5)

### Reconstruction of ancestral mutation haplotype

Of the 92 haplotypes that carried *NBN* c.657del5 and that could be deduced without ambiguity (S1 File), 22 (23.9%) carried identical alleles at all markers between the two most distant microsatellites D8S271 (allele 257) and D8S270 (allele 200). This haplotype (Table 8) was found in individuals from Poland, Germany, CS and Lusatia (Sorbs) and most likely represents the ancestral haplotype. The remaining 95 haplotypes from 40 homozygous and 15 heterozygous c.657del5 carriers (out of a total of 187 chromosomes) could not be resolved without ambiguity and are best guesses (S1 File). Among these haplotypes, we observed the putative ancestral haplotype not only in individuals from the countries mentioned above, but also from Bulgaria, Russia, and the Ukraine. Some 30% of chromosomes carrying the founder mutation showed the deduced founder haplotype (Table 8). Moreover, all 187 chromosomes carrying c.657del5 showed the same SNP alleles (Table 3). Therefore, the deletion is not only confined to individuals of Slavic origin but is most likely due to a single mutational event. All differences observed for individual microsatellite alleles are explicable by past recombination or mutation owing to the non-negligible recombination rate between the STR markers (Table 2) and the high mutation rates of microsatellites of 10^−4^ to 10^−2^ per generation.

**Table 8:** Illustration of past recombination events in *NBN* c.657del5 deletion carrying haplotypes.

Origin	D8S271	D8S273	InsDel	D8S1800	AFM289	H3GT	H2CA	D8S88	*NBN*	H5CA	D8S1811	D8S1724	D8S1146	D8S1618	D8S270
**aH**	**257**	**158**	**ins**	**145**	**189**	**216**	**232**	**90**	**c.657del5**	**189**	**108**	**142**	**258**	**274**	**200**
*Po*	**261**	**164**		**139**											
Po	266									**185**	**106**	**146**	**262**		**186**
Po											**82**	**138**	**258**		**196**
Po	**267**	**160**		**141**											
Po	**255**	**156**		**139**											
Po													**262**		**196**
Po										**185**	**106**	**136**	**262**		**186**
Po	**255**	**154**			**191**	**196**	**230**								
Po												**140**	**262**		**188**
Po	**265**			**139**						**185**	**106**	**140**	**264**		**186**
Po	**255**	**160**		**139**	**191**										
Po					**187**	**210**	**236**	**76**							
Po	**259**	**154**		**139**											
Po	**259**	**154**		**139**											
Po	**259**	**154**		**139**											
Po	**259**	**154**		**139**											
Po	**265**	**160**		**139**											
Po												**140**	**262**		**194**
Po												**140**			**192**
Po	**267**	**160**	**del**												
Po	**255**	**156**		**139**											
Cr	**255**	**156**		**139**										**286**	**186**
*Ru*	**255**	**160**		**139**											
*Ru*	**255**	**160**		**139**											
*Ru*	**259**			**139**	**191**						**100**	**136**	**268**		**194**
*Ru*	**265**	**160**		**139**	**191**										
*Ru*	**261**	**160**		**139**	**191**										
*Ru*	**261**	**160**		**139**	**187**	**208**									
*Ru*	**255**	**160**		**141**	**191**										
CS	**267**	**154**		**141**		**210**	**236**								
*CS*										**187**	**106**				**194**
*Uk*	**265**	**160**		**141**	**191**										
*Uk*	**265**	**160**		**141**	**191**										
*Uk*	**255**	**154**		**139**	**193**	**210**							**262**		**198**
*Uk*	**265**			**139**	**187**										
*Uk*	**265**	**154**		**139**											
*So*											**102**	**140**	**270**		**190**
*Bu*	**265**	**156**		**139**	**191**										
*Ge*	**263**	**154**		**141**	**191**	**210**	**236**	**86**							
*Ge*		**166**		**141**	**191**										
*Ge*	**265**	**154**													
*Ge*	**261**	**154**		**141**											
*Ge*	**255**	**154**		**141**	**191**										
*Ge*										**185**	**106**	**142**	**266**		**196**
*Ge*										**185**	**106**	**142**	**266**		**200**
*Ge*	**265**	**154**		**145**	**189**	**196**	**230**	**96**							
*Ge*	**261**	**156**		**139**	**191**								**262**		**186**
*Ge*	**255**	**162**		**143**											
*Ge*	**263**	**154**		**141**											
*Ge*	**261**	**154**		**139**											
*Ge*	**255**		**del**	**139**	**191**	**218**									
*Au*	**255**	**156**	**del**	**139**											
It		**166**		**139**	**191**	**214**									
Tu	261														194
Tu	261														194

**aH:** Reconstructed ancestral haplotype around the *NBN* c.657del5 mutation. The ancestral haplotype is based on the analysis of 13 microsatellite markers and one ins/del polymorphism (see Table 2). The 12 analyzed SNPs (Table 3) were located between markers D8S88 and D8S1146. Alleles deviating from the ancestral haplotype are specified. **Origin:** Po (Poland), Cr (Croatia), Ru (Russia), CS (Czech Republic/Slovakia), Uk (Ukraine), So (Sorbs), Bu (Bulgaria), Ge (Germany), Au (Austria), I (Italy), and Tu (Turkey). **Italicized origin:** haplotypes that could not be inferred without ambiguity. **Alleles in bold/shaded areas:** likely past recombination events, assuming a single event only (see main text for details).

We next aimed at inferring likely recombination events using an ad-hoc approach that postulated a recombination if two or more adjacent markers in the flanking regions of the original haplotype differed from the putative ancestral alleles. Using this approach, we identified more than 50 likely recombination events, most of them representing single events (Table 8). A rare deletion polymorphism was documented in one chromosome from Germany (255-158-del-139 at D8S271-D8S273-rs6150693-D8S1800) and one chromosome from Austria (255-156-del-139 at D8S271-D8S273-rs6150693-D8S1800) as well as in one Polish individual (267-160-del-145 at D8S271-D8S273-rs6150693-D8S1800). The flanking microsatellites on two chromosomes from Turkey shared the same alleles (261 at D8S271, 194 at D8S270) that were different from the original alleles (257 at D8S271, 200 at D8S270), rendering a common origin of the respective disease mutations rather likely.

## Discussion

We estimated the age of the *NBN* c.657del5 founder mutation and sought for an explanation of its widespread occurrence in Slavic populations. Our calculations place the original mutation at around 266 generations ago assuming a growth rate of 1% per generation, and at around 356 generations ago with a rate of 0.5%. These estimates translate into roughly 5300 to 7100 years, given a generation time of 20 years. These estimates are consistent with a previous assessment [14] of the age of the R1a Y-STR haplotype (4600 years), which is indicative of the Russian Plain branch (including proto-Slavic tribes) and which is present in Russia, Ukraine, Belarus, Poland and the Baltic countries. Furthermore, our estimates are in concordance with the proposed time of the split between the Proto-Balto-Slavic languages and the Indo-European tree around 4500–7000 YBP, but would predate the proposed split between Slavic and Baltic branches between 3500 and 2500 YBP (summarized in [12]). While this does not preclude the presence of *NBN* c.657del5 at low frequency in the steppe Yamnaya people before their admixture with Central and Western Europeans around 4500 YBP [46], our findings render far more likely a mutation origin in the ancestor population of all Slavic peoples, either before or after its admixture with the Yamnaya. Unfortunately, our data do not allow formal distinction between these two scenarios.

It should be noted that DMLE+ does not estimate the time to the most recent common ancestor (MRCA) of extant deletion carriers, but the actual mutational age. Thus, the deletion may have existed in the population at low frequency for some time before the MRCA. As regards the underlying population growth rate, we deem 1% to be a realistic assumption. While it is still possible that the true rate may have been closer to the (commonly used) value of 0.5%, rates of 0.1% or less appear unrealistic in view of the massive population expansion that took place during the late Neolithic and the Bronze Age. That different age estimates resulted from the use of two genetic maps is explicable in terms of slightly larger genetic distances in the Rutgers map, rendering recombination more likely and reducing the time span required to explain an observed haplotype pattern, compared to the deCODE map.

While the *NBN* c.657del5 mutation is found in all Slavic populations, a rather high heterozygote frequency (0.5–1.0%) has been reported for Ukraine (Eastern Slavs), Poland, CS, and Sorbs in Germany (Western Slavs), and for Bulgaria (Southern Slavs) (Table 9). These figures may still represent underestimates, because all of the countries are characterized by admixture between the Slavic and previous indigenous population. They also experienced massive population movement after World War II. Thus, while the heterozygote frequency was estimated to be 1 in 167 (9 of 1502) in Bulgaria when based upon a random collection of Guthrie cards, it increased notably to 1 in 111 (9 of 1002) when the analysis was confined to cards from individuals with Slavic surnames.

**Table 9:** Frequency of heterozygous *NBN* c.657del5 deletion carriers in different populations.

Country	Absolute number	Relative number	Allele frequency	Reference
**Poland**				
Mazowsze	10/1,620	1/162^a^	0.31%	Steffen et al. 2004[31]
Gdansk	21/4,000	1/190^a^	0.26%	Kanka et al. 2007[32]
Szczecin	3/530	1/176^b^	0.28%	Gorski et al. 2003[33]
Szczecin	9/1,500	1/167^a^^,^^b^	0.30%	Cybulski et al. 2004[34]
Malopolska	12/2,274	1/190^a^	0.26%	Varon et al. 2000[15]
Wielkopolska	16/2,090	1/131^a^	0.38%	Ziolkowska et al. 2006[35]
var. Voivodships	6/1,000	1/167^a^	0.30%	Chrzanowska et al. 2006[36]
**Czech Republic**				
	8/1,234	1/154^a^	0.33%	Varon et al. 2000[15]
	4/383 (34 y.)	1/96^b^	0.52%	Drabek et al. 2002[37]
	5/1,411	1/282^b^	0.18%	Pardini et al. 2009[38]
	2/915	1/457^b^	0.11%	Mateju et al. 2012[39]
**Ukraine**				
	5/908	1/182^a^	0.28%	Varon et al. 2000[15]
**Belarus**				
Minsk	1/1,014	1/1,014	0.05%	Bogdanova et al. 2008[40]
**Russia**				
	0/548	0/548^c^	0.09%	Resnick et al. 2003[41]
	2/348 (38.6 y.)	1/174^b^	0.29%	Buslov et al. 2005[42]
	0/344 (80.5 y.)	0/344^b^	0.15%	Buslov et al. 2005[42]
**Bulgaria**				
	9/1002	1/111^a^	0.45%	this report
**Croatia**				
	2/994	1/497^a^	0.10%	this report
**Slovakia**				
	3/2,996	1/999^a^	0.05%	Seemanova et al. 2004[43]
**Germany**				
not specified	1/886	1/886^b^	0.06%	Carlomagno et al. 1999[44]
Lusatia (Sorbs)	5/1,035	1/207^b^	0.24%	this report
Lusatia (Sorbs)	5/170	1/34^b^	1.49%	Maurer et al. 2010[16]
Northeast Bavaria	6/10,656	1/176^a^	0.28%	Maurer et al. 2010[16]
Berlin	1/990	1/990^a^	0.05%	Maurer et al. 2010[16]
Hannover	0/1,017	0/1,017^b^	0.05%	Bogdanova et al. 2008[40]
**China**				
	0/804	0/804^b^	0.06%	He et al. 2012[45]

Study design:

^**a**^ newborn;

^**b**^ adults (average age);

^**c**^ not specified.

The high frequency of c.657del5 carriers observed in Northeast Bavaria has been explained by local immigration of Slavic people between the 6^th^ and 9^th^ century [16]. On the other hand, the rather low heterozygote frequency in Slovakia was completely unexpected because of the high frequency of c.657del5 homozygotes, higher than in the adjacent Czech Republic. This discrepancy has been explained by the presence of traditional population isolates in Slovakia and by difficulties in collecting representative samples [43].

Transmission distortion has been put forward as an explanation of the abundance of c.657del5 (reviewed in [47]). Unfortunately, our data do not allow validation of this idea. Two other explanations for the persistence of a deleterious mutation in a population are chance (genetic drift) and reproductive advantage. Generally speaking, genetic drift explains well the increase in frequency of disease-causing mutations in local populations of recent origin, but does rather less convincingly so for a consistent trend across old and geographically separated populations [48]. On the other hand, balancing selection, involving a heterozygote advantage due to higher reproductive fitness, is a more likely explanation for the persistence of a deleterious founder mutation, thereby complementing the scarce evidence in humans (reviewed in [49]).

Based upon our study of the reproductive history of individuals from the Czech Republic, we found that female heterozygous *NBN* c.657del5 carriers gave birth to more children on average than non-carriers, pointing to a higher fertility of the former. No significant differences were observed between male carriers and non-carriers. Of note, our analyses of the number of offspring and of the reproductive histories partially included individuals from the same families. However, since we conditioned our analyses on mutation carrier status, observations in these analyses were conditionally independent and the applied statistical tests remained valid under the assumption of negligible intra-familial, i.e. block-like, effects other than mutation carrier-ship.

Clearly, many factors influence reproductive behaviour in modern societies. In our study, participants were interviewed before their *NBN* c.657del5 genotypes were known, and therefore the interviewer was blind to their *NBN* status. Moreover, the control homozygotes for the wild-type allele came from the same families. We found no evidence for differences between the two groups with respect to age at first menstrual period or at onset of menopause. However, we observed that the increase in offspring number was paralleled by a slight decrease in the number of spontaneous, not induced, abortions. Interestingly, a higher birth rate and lower spontaneous abortion frequency were also reported for female carriers of deleterious mutations in the DNA repair genes *BRCA1* und *BRCA2* [50–52]. From an evolutionary point of view, this link is explicable in terms of genetic variation in DNA repair genes that predisposes to cancer causing better survival under adverse environmental conditions [53].

The *NBN* gene is expressed in human germ cells. Immunofluorescent localisation showed that nibrin is specifically found at meiotic telomeres [54]. In all organisms tested so far, nibrin is required for meiotic recombination (reviewed in [55]). Moreover, it physically interacts with BRCA1 at the centrosomes and is obviously involved in mitotic centrosome maintenance [56]. Consequently, NBS patients show an impaired development of gonads and ovarian failure in females [2]. The analysis of a humanized *NBN* mouse model provides direct evidence, that the development of male germ cells is somewhat retarded while most female oocytes are arrested at pachytene[57].

Human female meiosis, but not male meiosis, is a highly error-prone process. Fewer than 30% of naturally fertilised human ova survive to term. The vast majority of losses occur before clinical recognition of pregnancy [58, 59]. The leading cause of these losses is a high rate of aneuploidies due to spontaneous non-disjunction during maternal meiosis [60–62]. Moreover, aneuploidies are also the major cause of spontaneous abortions in humans, affecting 10 to 15% of all recognized pregnancies. In principle, a slightly lower rate of non-disjunction in female carriers of the founder mutation could explain their higher fertility. Although highly speculative at this point, this assertion can be tested empirically.

The unusually high rate of human maternal meiotic non-disjunction and its further increase with age is explained by an interplay between unique features of oogenesis, i.e. recombination failure, loss of cohesion between sister chromatids, and sister centromeres in combination with a relaxed spindle checkpoint [63, 64]. This anomalous chromosomal behaviour still persists during the first mitotic divisions[65] and is paralleled by diminished mRNA levels of several checkpoint proteins and also of cohesin (reviewed in [66]). Carriers of the *NBN* c.657del5 mutation have a distinct gene expression phenotype with about the same number of genes up- and down regulated [67]. Moreover, based upon SDS-PAGE and mass spectrometry, it was shown that the two truncated proteins, p26 and p70, synthesized by carriers of the mutation, bind to proteins that do not interact with full-length nibrin [68]. It is tempting to speculate that these effects are also relevant in maternal germ cells and could explain the increase in fecundity of carriers. Thus, in future experiments, the interactors of these fragments should be studied in oocytes of females with the carrier mutation. In our view, the humanized *NBN* mouse would an ideal animal model to undertake such studies.

In summary, we presented evidence that the Slavic founder mutation *NBN* c.657del5, which has been implicated in Nijmegen Breakage Syndrome and cancer, may actually confer a reproductive advantage for female carriers due to higher fertility. Our age estimate for this mutation of less than 300 generations for this mutation implies that such an advantage could well explain the allele frequency of between 0.5% and 1.0% in Slavic populations.

## Supporting Information

S1 File Set of 92 haplotypes that could be inferred without ambiguity (lines 97–188) and that formed the basis for the age estimation of the *NBN* c.657del5 deletion plus 95 haplotypes that are best guesses (lines 2–96). (TXT)
